# Use of concurrent evaluation to improve implementation of a home fortification programme in Bangladesh: a methodological innovation

**DOI:** 10.1017/S1368980020000439

**Published:** 2021-04

**Authors:** Haribondhu Sarma, Md. Fakhar Uddin, Mohammad Ashraful Islam, Mahfuzur Rahman, Grant J Aaron, Catherine Harbour, Cathy Banwell, Tahmeed Ahmed

**Affiliations:** 1 Nutrition and Clinical Services Division, icddr,b, Mohakhali, Dhaka 1212, Bangladesh; 2 Research School of Population Health, Australian National University, Acton, ACT 2601, Australia; 3 Global Alliance for Improved Nutrition, Geneva, Switzerland; 4 Evidence, Measurement and Evaluation, The Children’s Investment Fund Foundation, London, UK

**Keywords:** Concurrent evaluation, Implementation science, Home fortification, Mixed method, Bangladesh

## Abstract

**Objective:**

This paper focuses on the use of ‘concurrent evaluation’ to evaluate a nationally scaled-up programme in Bangladesh that was implemented by BRAC (an international development organisation) using *Shasthya Shebika* (SS) – volunteer community health workers – to promote home fortification with micronutrient powders (MNP) for children under-five.

**Design:**

We developed a programme impact pathway to conceptualise the implementation and evaluation strategy and developed a strategic partnership among the key programme stakeholders for better use of evaluation evidence. We developed a multi-method concurrent evaluation strategy to provide insights into the BRAC programme and created provision for course correction to the implementation plan while it was in operation.

**Setting:**

One hundred sixty-four sub-districts and six urban slums in Bangladesh.

**Participants:**

Caregivers of children 6–59 months, SS and BRAC’s staff members.

**Results:**

The evaluation identified low awareness about home fortification among caregivers, inadequate supply and frequent MNP stockouts, and inadequate skills of BRAC’s SS to promote MNP at the community level as hindrances to the achievement of programme goals. The partners regularly discussed evaluation results during and after implementation activities to assess progress in programme coverage and any needs for modification. BRAC initiated a series of corrections to the original implementation plan to address these challenges, which improved the design of the MNP programme; this resulted in enhanced programme outcomes.

**Conclusions:**

Concurrent evaluation is an innovative approach to evaluate complex real-world programmes. Here it was utilised in implementing a large-scale nutrition programme to measure implementation process and effectiveness.

Identifying an appropriate evaluation approach is essential to understanding the implementation process, particularly to gain insight into the ‘black box’ of what happens during the implementation period^(1,2)^. The implementation, analysis framework and interpretability of results of any evaluation at a national scale are often complex. For example, when an intervention is implemented in a complex setting, a number of factors might influence it independently or interdependently^(1,3,4)^, which affects the impact evaluation design approach and potential interpretability of results^(5)^. Considering this, implementation researchers suggest evaluating the implementation process prospectively instead of retrospectively, alongside the outcome evaluation, to get a comprehensive understanding about what occurs during implementation^(1,6)^, as well as to create opportunities for course correction.

There are numerous types of evaluations available for assessing any given intervention. Many traditional evaluations took a summative approach^(7)^ and measured the outcome of the programme, but did not assess how this outcome occurred. These evaluations are quantitative^(8)^ and measure associations between inputs and outcomes using statistical methods. Traditional evaluation usually relies on a pre–post assessment strategy, conducting baseline and endline assessment and measuring the outcome or impact of the programme at programme end. One of the main limitations of this approach is that it cannot clarify explicitly why certain outcomes occur, which limits the utility of the data for providing programme feedback tools for decision-makers and implementers to transform that knowledge into action – that is, programme course correction enhancements^(9,10)^.

To address these challenges, there are several other alternative approaches, such as formative evaluations^(11)^, real-time evaluations^(12)^, developmental evaluations^(13)^ and concurrent evaluations^(14)^. In this paper, we focus on a concurrent evaluation design – an innovative evaluation strategy that synthesises multiple evaluation methodologies and which can be used to improve both the implementation and outcome of a programme. We applied this evaluation approach to a home fortification or point-of-use fortification programme in Bangladesh that targeted infants and young children with micronutrient powders (MNP). According to the WHO, home fortification or point-of-use fortification of foods with MNP has been effective in improving the vitamin and mineral intake of infants and young children in low-income settings^(15)^. There is now substantive evidence on the efficacy of MNP; however, there are far fewer examples of programme effectiveness and impact. The findings of our research team’s evaluation activities have been split into several manuscripts and published as part of this supplement. This paper focuses primarily on methods and secondarily on overall evaluation findings, particularly those that had implications for course correction during programme implementation.

As the name suggests, a concurrent evaluation is implemented at the same time as programme implementation as a means to assess its progress, thus determining both how a programme works and whom it benefits. To make necessary course corrections, data collection, analysis and reporting must take place during the programme implementation period, and there must be frequent feedback loops to programme implementers and decision-makers^(14)^. Concurrent evaluations use a mixed-method approach and can be an innovative way to provide actionable insights to the programme.

## Concurrent evaluation of BRAC’s home fortification programme

In Bangladesh, BRAC, a non-governmental organisation, implemented a large community-based programme on home fortification of foods with MNP (locally branded as Pushtikona-5) from 2014 to 2018. The programme aimed to reduce the prevalence of Fe deficiency anaemia (IDA) among children of 6–59 months. BRAC implemented the programme nationally in 164 rural sub-districts and in 6 urban slums in Bangladesh. The programme specifically targeted the use of MNP among children aged 6–59 months by a cadre of female volunteer community health workers (CHW) called *Shasthya Shebika* (SS) and paid CHW called *Shasthya Kormi* (SK). In Bangladesh, the sexes are usually publicly separated and gendered roles strictly adhered to; women (mothers or close relatives) are designated as children’s primary caregivers. Female health workers, therefore, have greater access to female primary caregivers compared to male health workers. Therefore, BRAC recruited both SS and SK from female community members. A detailed description of the programme design and implementation strategy is available in online supplementary material, Supplemental file 1.

During the design phase, the programme implementers and donor requested that the evaluation strategy: (1) address real-world constraints in the programme; (2) facilitate course correction decisions during programme implementation to improve programme effectiveness; and (3) evaluate the overall programme impacts on anaemia reduction. This paper is a part of a supplement of Nutrition Implementation Science and describes the concurrent evaluation design used for BRAC home fortification with MNP. This article shares the evaluators’ experiences with providing insights into the BRAC programme and recommendations for course correction to the implementation plan while in operation.

## Methods

### Programme impact pathway for home fortification with micronutrient powders

**Fig. 1:**
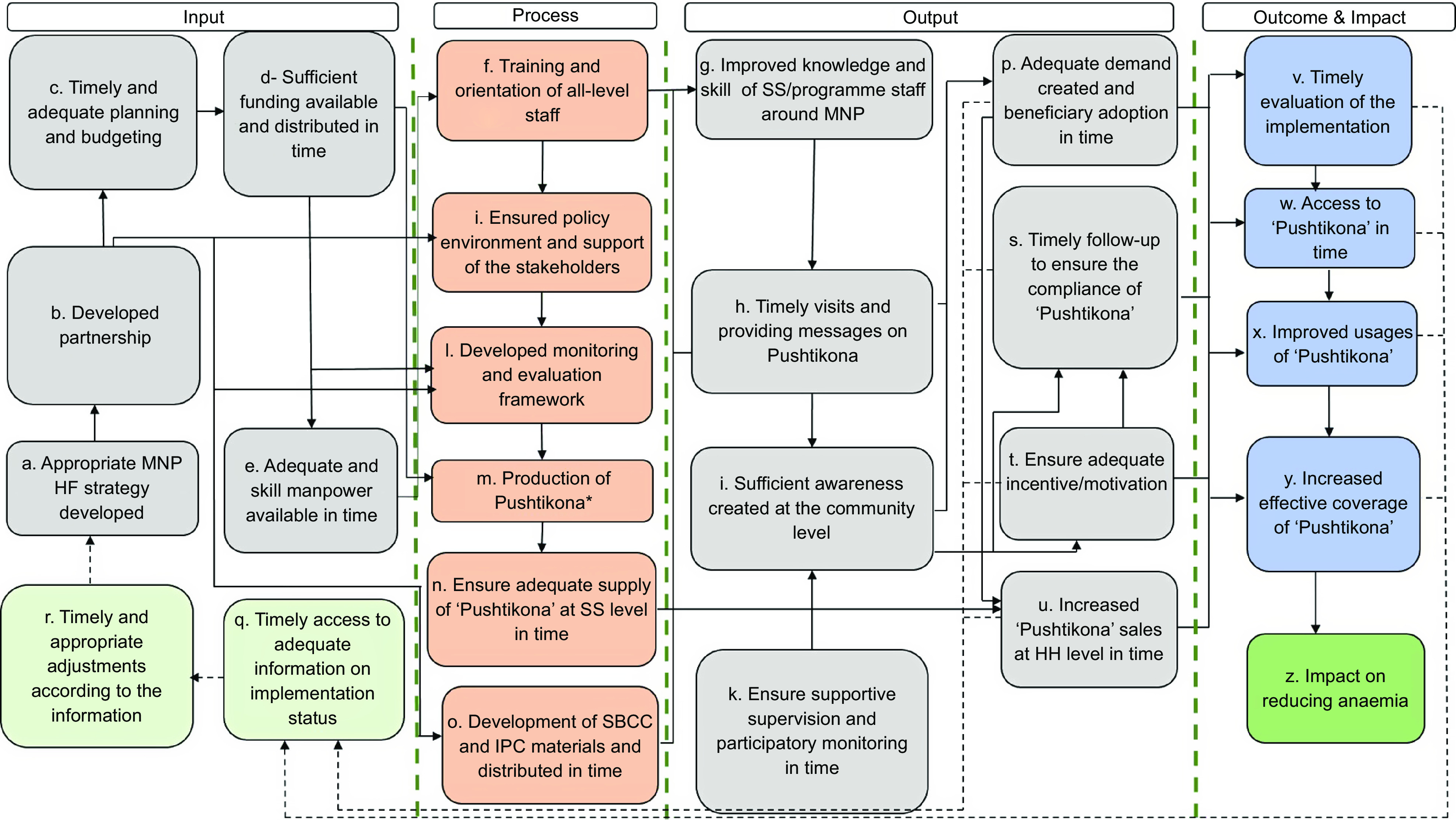
Programme impact pathway of BRAC’s home fortification programme in Bangladesh

Programme impact pathway (PIP) is a process to theoretically conceive the whole programme implementation, connecting programmatic inputs from delivery through household and individual utilisation and impact, taking into consideration the contextual factors that might influence the effectiveness of implementation^(16)^. We developed a PIP framework (Fig. 1) in consultation with partners and other relevant stakeholders of the programme and based on document review to conceptualise the implementation and evaluation design of BRAC’s home fortification programme. The impact pathway determined what steps had to be followed in the programme to reduce IDA, and this further guided the evaluation from its design through analytical framework.

*Pushtikona is the brand name of a micronutrient powder of BRAC.

As shown in Fig. 1, PIP illustrates the key milestones of BRAC’s home fortification programme (denoted alphabetically from a to z in Fig. 1). We considered the successful implementation of programme milestones a precondition to achieve the expected outcomes and impact of the programme. The impact of a successful programme implementation reduced anaemia among the targeted children as illustrated in the final milestone (z) denoted under outcome and impact of PIP (Fig. 1). In general, PIP conveys assumptions about the preconditions for change to occur. In the evaluation context, PIP shows the organisation of the process around high-level milestones. For example, an appropriate home fortification with MNP was initially developed with partnership among donors, implementers, knowledge brokers and evaluators (a, b). Partnerships were developed to create an enabling environment, develop communication materials and implement the intervention in time with proper technical assistance and guidance (j, n). The development of an adequate and comprehensive implementation plan and budget was needed to ensure availability of required funds and start implementation in time (c, d). Simultaneously, the estimated number of workforce (all-level staff) was planned to be recruited and trained to perform quality implementation activities (e).

Under the process and output of PIP, training, education and motivation of BRAC SS were essential to ensure adequate delivery of messages to caregivers and to clarify their queries relating to MNP so as to create demand and increase sales and coverage of MNP (f–i, p, s–u). Moreover, the home fortification programme engaged key leaders and other stakeholders at the district, sub-district and national levels to have their support, raise awareness and build their understanding of MNP (j). PIP illustrates that supportive monitoring and supervision of programme staff can increase the sales of MNP at the household level, which may eventually increase the coverage of MNP (k, u, s, x, y) denoted under output and outcome of PIP. Household visits of SS on time can ensure caregivers’ compliance with the use of MNP. Incentives to the SS can motivate them to perform home visit activities as planned. The coverage of MNP was anticipated to increase through timely demand generation, supply, improved knowledge and motivation of SS, home visit and incentives. Timely production and supply of MNP from the manufacturer to BRAC and from BRAC headquarters to the sub-national level were also expected if sufficient funds were available and requisition of products were placed on time (d, m, n). Continuous and consistent supply of MNP sachets at the SS level was essential to ensure increased and consistent sales of MNP (u).

Besides the advancement of these milestones, timely evaluation of its process, activities and achievements was required for further course correction and appropriate adjustment in programme strategy in time (p, s–y, q, r), denoted under the output and outcome of PIP. Therefore, the milestones needed to be assessed through exploring the status of outputs and outcomes (e.g., increased sales of MNP at the household level, adequate demand created in the community level and increased coverage of MNP). Timely access to adequate information on implementation status may help stakeholders to take timely and appropriate adjustment, which is considered a management feedback loop in the pathway framework (q, r, a). In this way, PIP demonstrated a feasible pathway to reduce anaemia.

### Activities of concurrent evaluation

**Fig. 2:**
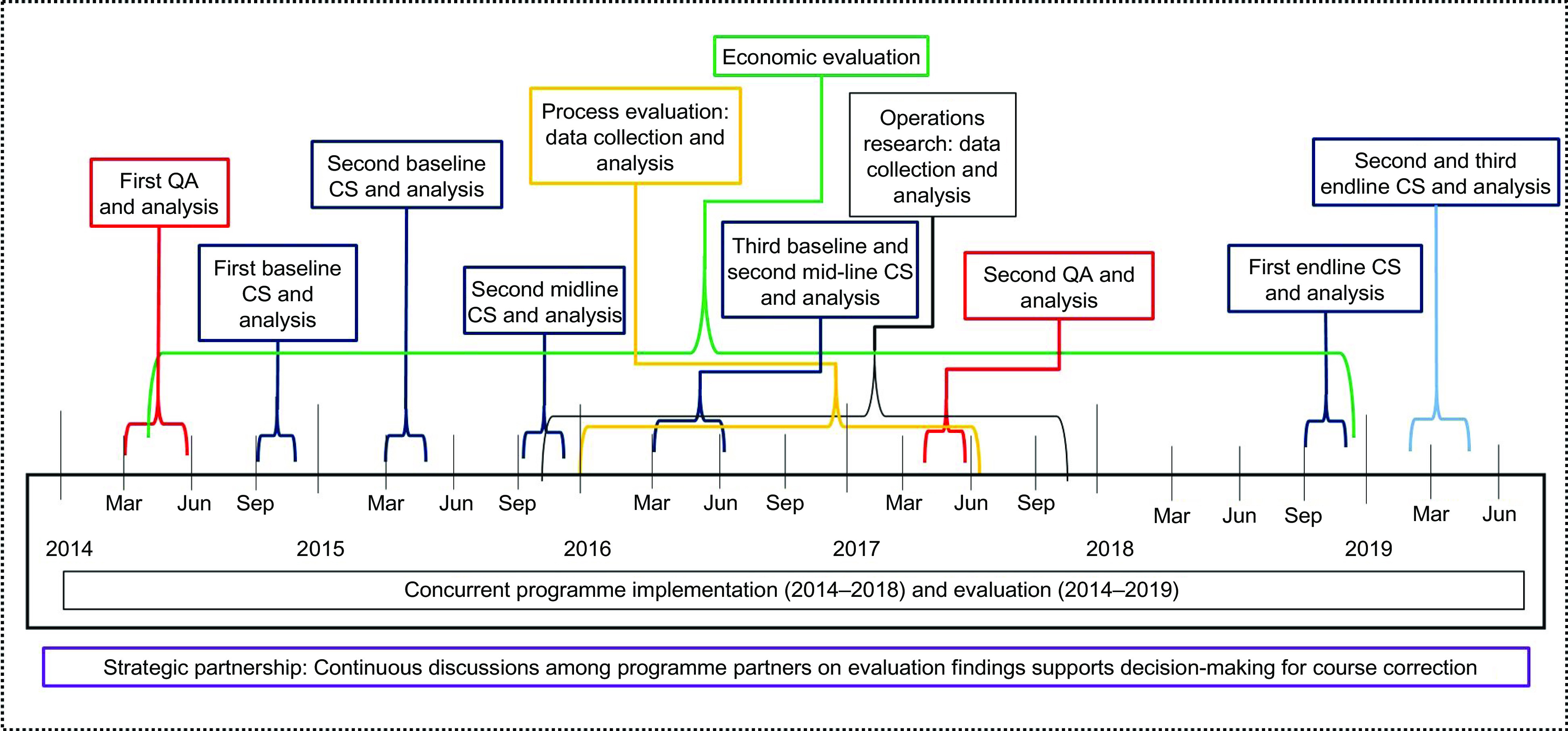
Implementation and timeline of concurrent evaluation activities. QA, quality assessment; CS, coverage survey

Our concurrent evaluation used mixed-methods approaches that included both quantitative and qualitative assessments of activities, outcomes and impact of the programme throughout the implementation period. Quantitative assessment was designed as a series of surveys to assess the coverage of MNP and impact of the programme on the reduction of anaemia. The qualitative part of this evaluation was designed to cover the nature and scope of participatory evaluation, which was implemented through concurrent qualitative data collection. Additionally, operations research and cost-effectiveness analysis were carried out to provide additional evidence that helped in reaching the intended goals of strengthening project performance during implementation. Evaluation activities were implemented sequentially and in three phases, corresponding to the three phases of programme implementation. Figure 2 shows evaluation activities and timelines of concurrent evaluation.

### Coverage surveys

We conducted eight cross-sectional surveys; three of these were baseline surveys, and three were their corresponding endline surveys in three programme platforms (see online supplementary material, Supplemental file 1), and two were midline surveys in the first two programme platforms. In the first two programme platforms, midline surveys were implemented 12 months after the baseline surveys, while endline surveys were implemented 36 months after the baseline surveys. In the third platform, an endline survey was done 2 years after the baseline survey. Baseline, midline and endline surveys were implemented at approximately the same period of the year in each platform to control for potential effects of seasonality. Details about the implementation of coverage surveys, including sample size, sampling, covariates, outcome variables, data collection procedures and data analysis, are described in an unpublished manuscript.

### Concurrent qualitative data collection

As part of the mixed-methods approach, qualitative data collection was used to provide an in-depth description, analysis of implementation processes and patterns of interactions with other contextual factors of implementation. The aim of these integrated approaches was to provide flexibility to fill in gaps in the available information, to use triangulation to strengthen the validity of the estimates, and to provide different perspectives on complex, contextual and multidimensional phenomena around the implementation of BRAC’s home fortification intervention. The evaluation team conducted key informant interviews, in-depth interviews, focus group discussions (FGD) and observed child feeding practices with a view to attaining different objectives during different phases of the evaluation. Table 1 shows the timeline, objectives, study participants and techniques applied in qualitative assessments. The first qualitative assessment assessed the knowledge, attitudes and practices of caregivers of children aged 6–59 months and those of SS and SK of BRAC about child nutrition, home fortification with MNP and also explored the satisfaction and motivation of SS towards their voluntary work. The second qualitative study explored the underlying factors that related to achieving/failing to fulfil the sales target for MNP in BRAC’s home fortification programme areas.

**Table 1:** Timeline, objectives, study participants and techniques applied in qualitative assessments

Qualitative assessment	Study period	Objectives	Study participants	Data collection technique
Assessment 1	March 2014–August 2014	(i) To assess the knowledge, attitudes and practices of caregivers of under-five children about MNP and child nutrition (ii) To assess the knowledge, attitudes and practices of SS and SK	(i) Primary caregivers of under-five children (ii) Fathers of under-five children (iii) Officials from BRAC at the sub-district level (iv) Upazila (Sub-district) Health and Family Planning Officer	(i) Key informant interview (ii) In-depth interview (iii) FGD (iv) Observation
Assessment 2	October 2016–January 2017	(i) To explore the underlying factors relating to achieving/failing to fulfil the sales target of MNP in BRAC’s home fortification programme areas (ii) To explore the underlying factors relating to low coverage of MNP	(i) Frontline health workers (SS, SK) of BRAC (ii) Primary caregivers of under-five children (iii) BRAC’s field organisers (iv) Other stakeholders (i.e., private practitioners and health workers from public sector and development workers in the catchment areas of the study)	(i) Key informant interview (ii) In-depth interview (iii) FGD (iv) Informal discussion

### Process evaluation

We considered process evaluation as a key activity under concurrent evaluation. Concurrent evaluation considers both process and outcome indicators, as well as the overall assessment of programme effectiveness^(17)^. Whereas, process evaluation designs to assess process indicators generally^(18)^. The overall aim of process evaluation was to assist implementers of the home fortification programme to attain a clear understanding of whether the intervention was being implemented as planned or not, and to provide the opportunity to refine the implementation strategy and action plans prior to measuring the outcome and impact of the intervention. Process evaluation was designed as a prospective evaluation; however, this evaluation collected data both retrospectively and prospectively to capture the full range of the implementation process. The current study was designed to follow two investigation steps: process tracking and in-depth investigation.

We performed process tracking to understand the implementation process and whether or not the intervention was implemented as planned. These activities identified the key programmatic successes and challenges in the existing implementation process for root-cause analysis and in-depth qualitative investigation. The identified programmatic challenges and successes from the initial findings of process tracking were shared with the national-level stakeholders of partner institutes, including BRAC. Participants of the meeting helped identify and prioritise the areas of in-depth investigation and root-cause analysis that led the process evaluation team to develop some research questions.

We conducted in-depth investigations to identify the underlying factors of key challenges and successes. We conducted key informant interviews, in-depth interviews and FGD with key stakeholders of the programme at national and sub-national levels. We observed BRAC’s training sessions on home fortification with MNP provided to SS and SK. We reviewed documents relating to the home fortification programme, such as programme register, MNP calendar, quarterly progress report of BRAC, implementation plan for Social Behavioural and Communication Channel (SBCC), interpersonal communication materials, monthly sales reports of BRAC, as well as guidelines and instructions of bimonthly special refresher training.

### Operations research

At the beginning of year 2 of implementation of the home fortification programme, based on an initial analysis of qualitative and coverage survey data, review of available documents and discussion with key stakeholders, including representatives of partners and the donor, we formulated an operations research question with the aim of improving programme delivery, which was: Why do the SS have less contact with households with children <2 years of age and how to increase their home contact?

To answer this question, first, we analysed data on initial qualitative assessments, then conducted a mixed-methods baseline assessment as part of this operations research. Findings of these assessments helped us identify the challenges BRAC’s SS faced in implementing home fortification interventions at the community level. To address these challenges and to increase home contact of BRAC’s SS, we designed a quasi-experimental study comprising four arms: three intervention arms and one control arm. BRAC’s existing interventions were implemented in all the intervention arms. In intervention arm 3, we added some additional interventions considering the gaps identified through initial assessments. Periodic assessments were done in designing and revising intervention activities, giving feedback to course correction of BRAC’s home fortification programme and, finally, assessing the outcomes and impacts of the intervention.

### Economic evaluation

Economic evaluation of the MIYCN home fortification programme aimed to estimate the start-up cost and implementation cost of the programme and the cost per anaemia case averted. Activity-based costing was applied for calculating the cost of MIYCN in both start-up and implementation phases. The key outcome indicator was calculated as the total number of anaemia cases averted and total disability-adjusted life years (DALY) averted. The outcome of the programme was estimated using change in the prevalence of anaemia between baseline and endline periods (coverage survey).

### Data analysis, synthesis and reporting

We designed separate analysis plans to answer specific research questions. For example, to understand the barriers in the home fortification programme hierarchy associated with home visit by SS of BRAC, we applied multi-level modelling. In this way, data from different periodic assessments were analysed, synthesised, and estimation of the effect of intervention on the primary outcome of the programme was done. A detailed description of quantitative analysis will be published separately. Quantitative findings were triangulated with qualitative findings collected through concurrent qualitative assessments. After synthesising the findings from different evaluation components, we shared those with the stakeholders for timely course correction of the programme. We performed thematic analysis for qualitative data; a detailed description of the analysis of qualitative data has been reported in another paper^(19)^.

### Synergies and partnership around evaluation

**Fig. 3:**
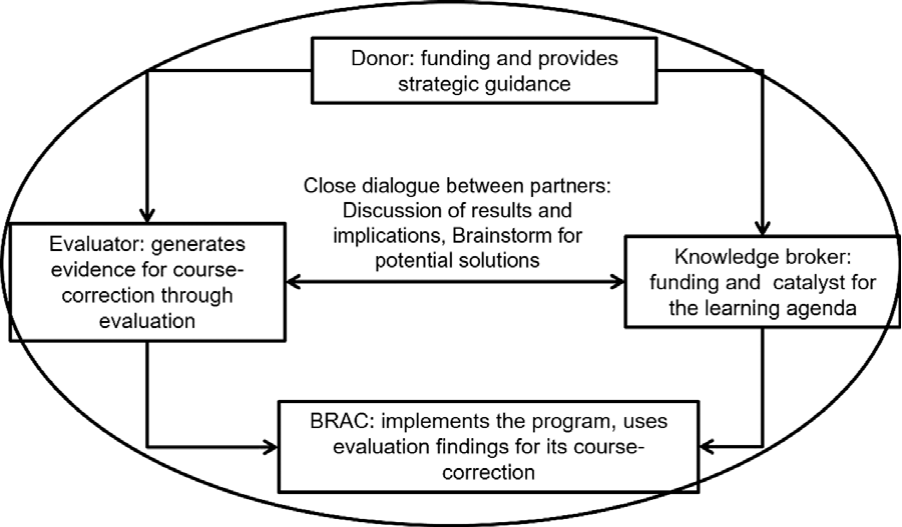
Synergies and partnership around concurrent evaluation

For timely course correction, this evaluation created a strategic synergy around partnership among the implementer (BRAC), evaluator (icddr,b), donor (e.g., funder – The Children’s Investment Fund Foundation, UK) and knowledge broker (Global Alliance for Improved Nutrition, creates connections between the evaluator and implementer)^(20,21)^. All the partners had specific roles in programme implementation. Figure 3 illustrates the synergies and interactions among all partners. As a funding organisation, the donor played a stimulating role through providing strategic guidance for a successful programme implementation at scale in order to achieve its expected outcomes and impact. They adopted an approach to using actionable data and evidence throughout the period of programme implementation, which came from concurrent evaluation research. The donor, therefore, hired an external evaluator for conducting the evaluation to generate evidence for timely course correction. The donor was assigned to the knowledge broker to provide technical assistance to the implementing partner for a successful programme implementation and to maintain a close relationship with the evaluator for its evaluation activities.

At the national level, the knowledge broker was responsible for building a synergy among the donor, evaluator and implementer through regular close dialogues in order to ensure the usage of evaluation findings for timely course correction of the programme by the implementers. However, this synergy and partnership approach around concurrent evaluation was planned for a timely identification of barriers and facilitators relating to programme implementation and coverage of MNP, which helped the programme to be on track. Robust evidence coming from the evaluation ultimately helped implementers to make a strategic revision in their programme to achieve the expected outcomes and impact at scale. In our capacity as evaluator, we conducted qualitative and quantitative assessments at different time-points during programme implementation. We shared the findings with the implementers, knowledge broker and donor representatives through meetings, dissemination and reports immediately after conducting each of the assessments to make course corrections in time (Fig. 3).

## Results

### Evaluation activities and course correction

Table 2 describes the evidence generated from different evaluation studies and BRAC’s course corrections in 2014–2016. The implementers made extensive use of the findings from concurrent evaluation through programmatic course correction to achieve improved sale and coverage of MNP. We rationalised the need for course corrections in the existing programme at different implementation time-points. Through a demonstration of the findings in the programmatic context, a strong methodological justification for course corrections was made by BRAC as an implementer.

**Table 2:** Evaluation activities, evidence and course correction in the implementation plan by BRAC

Evaluation activities 2014–2018	Evidence from findings of evaluation	Course correction by BRAC
First qualitative assessment, 2014	CHW sold MNP on loan, and sometimes distributed MNP sachets free of charge	Provided four boxes of MNP free of charge to the CHW as revolving fund
	Unwillingness of family members to buy MNP	Developed simple pictorial counselling card for use by frontline workers for interactive counselling with caregivers; the same materials were also used in social mobilisation sessions
	Limited storage capacity resulted in stock-outs of MNP at the national level	Hired warehouse spaces at both central and regional levels
	Mothers who had interactions with BRAC’s CHW were more aware of MNP	Increase in the incentives for CHW to increase their contact with mothers
First baseline survey, 2014 in phase I districts	Limited home visits by BRAC’s CHW: only 25 % of households had been visited within 2 months of the survey	Changed the incentive disbursement period and provided micro-business plan training to CHW
		Recommendation to conduct operations research to improve the performance of CHW
Second qualitative assessment, 2015	CHW’ lack of skills in promoting MNP	Revised refresher training strategy
	SK/PK were demotivated to assist the SS when only the SS were eligible to receive incentives	Introduced performance-based incentives for SK/PK
	Stock-outs of MNP among CHW and households: supplies of MNP were not increasing although demand had increased	Ensured the availability of buffer stocks at the sub-district level
Second baseline survey, 2015 in phase II districts	72 % of caregivers reported having run out of MNP in their households	
First midline survey, 2015 in phase I districts	Limited use of MNP calendar at the household level (only 4·1 % of households had an MNP calendar and, among these households, only 2·5 % marked on the calendar)	Developed, tested and distributed an easy-to-use MNP card considering the challenges in using a calendar
Operations research, 2016	CHW lack confidence in promoting MNP to their communities ‘We are either illiterate or have only basic education. So, why should people listen to us?’ (CHW, FGD)	Revised recruitment criteria of BRAC’s CHW
		Created counselling card (job-aid) for CHW to assist them with counselling so that all caregivers would receive a consistent message
Process evaluation, 2016	Coordination gap between the sub-district and national-level programme staff in terms of understanding the demand and stock of MNP	Recruited supply chain officer to monitor and maintain supply and stock issues and to conduct record-keeping and reporting

The first qualitative assessment conducted in 2014 identified some challenges at the national and sub-national levels relating to the implementation of home fortification programme. MNP were not available at BRAC offices at the sub-national level to distribute among SS due to inconsistent MNP supply from BRAC headquarters because of stock-out of MNP at the BRAC central warehouse due to its limited storage capacity. After a recommendation based on this finding, BRAC hired a separate central warehouse to reduce the problem of MNP stock-out, which later ensured consistent MNP supply from national to the sub-national level.

Other barriers in programme implementation identified through qualitative assessments included: (i) caregivers were not aware and motivated enough to purchase MNP due to a lack of interaction between caregivers and CHW, (ii) social mobilisation activities were inadequate, and (iii) SS had to sell MNP on credit and, sometimes, they distributed MNP sachets free of charge as a sample to generate demand. In addition to these qualitative findings, the first baseline coverage survey conducted in mid-2014 reported low household visits by the SS. To address these challenges, course corrections were made by BRAC, such as revision in incentives for SS, a revolving fund for SS (four boxes of MNP) and distribution of social mobilisation brochures. BRAC also drafted an income-generating guideline for the SS to improve their home visits. Additionally, findings from the evaluation recommended conducting an operations research to address the problem of SS’s home visits and improve their performance in selling MNP.

The second qualitative assessment conducted in 2015 revealed an increased demand for MNP at the community level, but still there remained some programmatic challenges. Stock-out of MNP was found at the SS level, and they could not be distributed despite having demand from beneficiaries. The second baseline survey done in 2015 reported that 72 % of caregivers reported run-outs of MNP at their households. This happened due to a lack of buffer stock of MNP at BRAC’s local offices. This occurred because of interruption in MNP supply from BRAC headquarters to the sub-national level due to political instability and inadequate transport facilities. The evaluators shared the findings with BRAC and recommended that the problem of MNP stock-out be addressed at the sub-national level to ensure consistent MNP supply from local offices of BRAC to the SS. Considering the recommendation, BRAC then maintained buffer stocks at local offices at least for the subsequent 2 months.

Bimonthly special refresher training was not as effective as expected in building the capacity of CHW in some sub-districts due to inadequate discussions held on home fortification, inefficient time management and insufficient participation. The absence of focal persons of the programme, insufficient conveyance bill for using local transportation and lack of communication were identified as underlying causes of the challenges relating to bimonthly training. The evaluation recommended that participation of all categories of SS (e.g., high-performing, low-performing) should be included in refresher training, and focal persons of the MIYCN programme should be involved as facilitators.

Lack of skills in carrying out home fortification activities was found among the SS due to sub-optimal monitoring by the SK during their follow-up visits in building SS’s capacity at the community level. We recommended introducing incentives for the SK to get them more involved. Accordingly, BRAC arranged incentives for SK (if an SK could achieve up to 80 % of the target on sales, she would get BDT 300 (1 BDT = 0·012 USD) as an incentive).

Operations research conducted in 2016 reported a lack of confidence among the SS in providing counselling to beneficiaries. The current study recommended developing an income generation guidance for the SS with a set of skill-based guidance to the programme, including revision of the recruitment criteria for the SS considering their age, education and development of a job-aid. Accordingly, BRAC took initiatives to address these recommendations. Subsequent evaluation activities also revealed that this job-aid to CHW ensured that as many caregivers received a unique message about MNP.

The second concurrent qualitative assessment identified interruptions in the flow of information relating to a timely requisition of MNP supply from sub-national to the central level. Demand for MNP was placed from sub-district levels to BRAC headquarters, with an unanticipated amount due to coordination gaps between sub-district and national-level staff. Besides, the monthly targeted volume of MNP was not distributed as local health managers perceived that it could increase the workload of SS and might hamper other programme activities of BRAC. Therefore, BRAC made a decision to recruit a focal person – Officer Supply Chain & Quality Assurance (OSCQA) – whose role was to identify the problems relating to MNP stock-out at the sub-national level, ensure timely requisition and consistent supply of MNP through regular field-visits and regularly communicate with the programme staff of BRAC at the headquarters. Although recruited in March 2016, the OSCQA began to ensure a smooth supply of MNP from the central to sub-national levels from the third year of its implementation, and no stock-out of MNP has ever since been reported.

### Outcome of course corrections: increased sale of micronutrient powders sachets

**Fig. 4:**
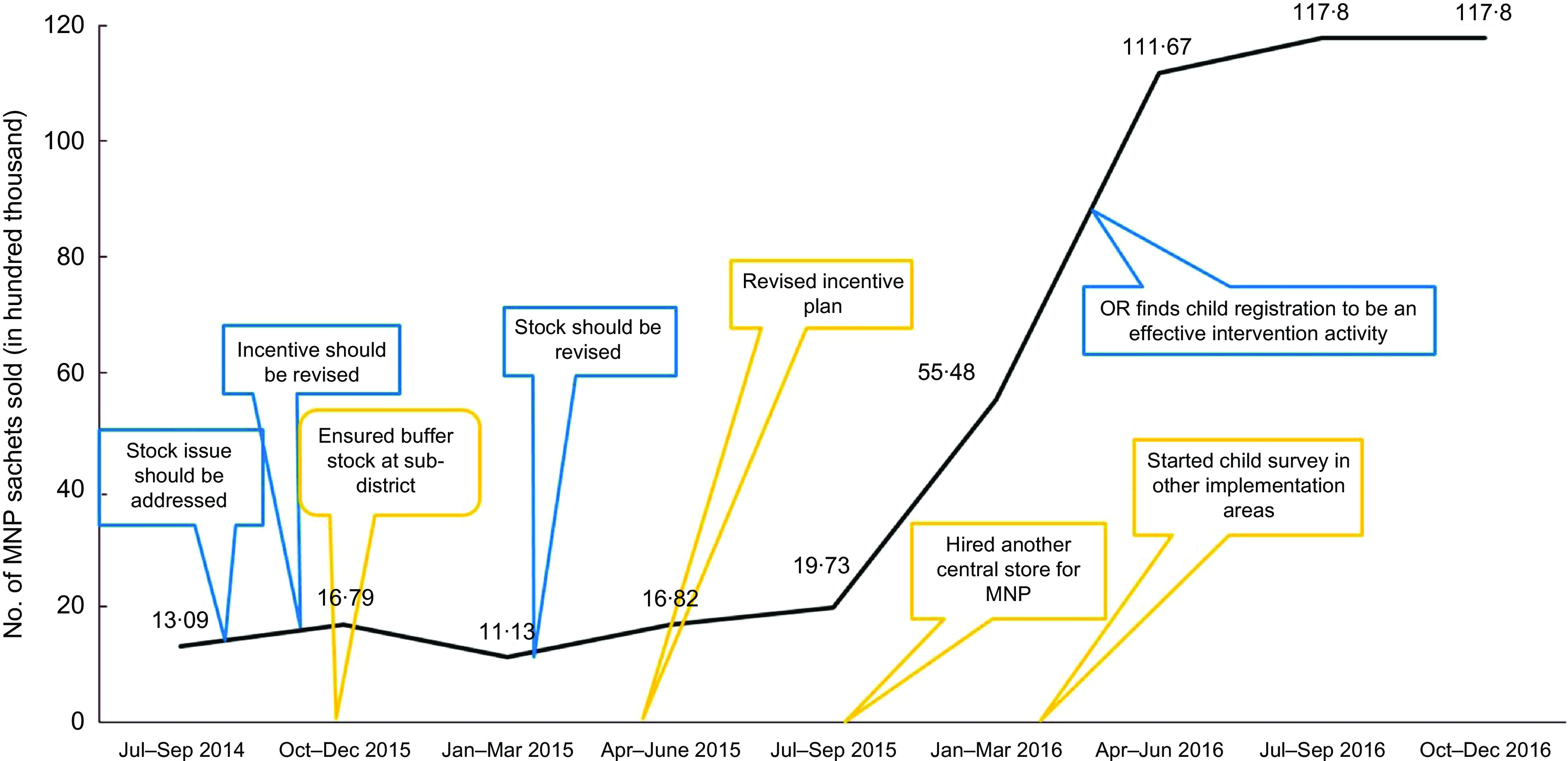
Increased sale of MNP sachets after addressing implementation bottlenecks and gaps based on evaluation findings. We analysed data on BRAC’s sale of MNP to measure a periodic sale trend and triangulated this trend with different evaluation events. The line indicates the sale of MNP sachets at different time-points of implementation. The blue box indicates recommendations made, based on evaluation findings at different time-points of implementation. The green box indicates course correction initiatives by BRAC at different time-points of implementation. MNP, micronutrient powders

One of the main outcomes of these course correction initiatives was a dramatic improvement in the trends of selling MNP sachets. Figure 4 illustrates a significant increase in sales as a result of addressing several implementation bottlenecks and gaps identified by concurrent evaluation. Prior to course correction measures, the sale of MNP remained very low with a trend at around two million MNP sachets during first years of programme implementation; however, sales increased dramatically to more than 10 million sachets after a year (Fig. 4).

## Discussion

### Implications of concurrent evaluation in implementation science relating to nutrition interventions

Implementation science literature suggests systematically identifying implementation bottlenecks and gaps and developing timely implementation strategies^(22–24)^. Using the findings of an evaluation for a timely course correction is critical for a successful implementation of any nutrition intervention in a complex real-world setting. This paper demonstrated how a concurrent evaluation can be implemented simultaneously, alongside the implementation of a home fortification programme and improved implementation fidelity through real-time course corrections of implementation plan.

As is inherent in the nature of a complex intervention^(25)^, BRAC’s home fortification programme also was embedded with many features since the programme was scaling up nationally and was being implemented in a real-world setting where uncertainty continuously threatened programme implementation. For these reasons, the evaluation was designed to accommodate such uncertainties. Our evaluation methods were grounded with comprehensive, complex approaches^(26)^; developed a PIP; formed strategic partnerships with all groups involved in the programme; considered the contexts of the MIYCN programme in account; and gathered detailed pieces of evidence into an accurate estimation of investments, efforts, process and outcomes.

Concurrent evaluation improved the accessibility and utilisation of MNP at beneficiary levels. Evidence-based course correction increased MNP sales, which ultimately improved access and utilisation of MNP among caregivers. In a market-based MNP intervention, caregivers buy MNP products for their children; they are more likely to use it^(27)^, which would eventually improve programme usability at the community level. A previous study conducted in a low-income setting suggested that the sale of MNP is an effective means to improve usability as well as reduce IDA among young children^(28)^. Market-based public health interventions generally have greater effectiveness and sustainability compared with free distribution strategies^(26,27)^. This paper demonstrated that concurrent evaluation could be used as a tool to improve programme implementation and utilisation.

Concurrent evaluation documented the entire programme implementation process clearly, including the rise in MNP sales, implementation gaps and course correction activities. This institutional knowledge management process will be used when the intervention is scaled up further, and to sustain the MNP programme for longer periods. The experiences in using evaluation evidence for addressing implementation bottlenecks and gaps are critical for future implementation of home fortification or similar interventions. Evidence from this concurrent evaluation, in addition to helping BRAC’s home fortification programme, may also serve as a valuable reference to implementation researchers and policy-makers in other contexts. These lessons learnt also give a direction about how an intervention can be sustained for a longer period and can promote dissemination of findings into other settings^(11)^.

### Strengths, limitations and future considerations

Strengths of this evaluation include robust quantitative and qualitative research methods, rapid turnaround from data collection to reporting, and independence of evaluation from the implementation process while maintaining frequent programme feedback loops. Due to the complex nature of the programme, there were no single existing research methods that could be applied directly for the evaluation. Our approach relied on evaluation experiences of a broad team of researchers from various disciplines. Cross-disciplinary thinking is a valuable resource for concurrent evaluation, particularly in complex programmes like the one discussed in the current paper.

The evaluation methods we used for BRAC’s home fortification programme may be used carefully in other settings when implementing concurrent evaluation. Methodological innovation and flexibility in identifying appropriate methods are the key characteristics of concurrent evaluation. Researchers of concurrent evaluation may find more suitable methods and tools considering the contexts of implementation. Researchers also should emphasise building capacity to understand the methodologies and should consider the challenges of concurrent evaluation while designing future evaluation studies.

In order for the results of a concurrent evaluation to have maximal impacts on programme course correction, it is important that there is a clear understanding among all parties of the role of the evaluator. Historically, programme evaluations have focused solely on pre–post evaluations with complete independence of evaluators from implementers. While a case could be made that this strengthens the validity of the findings, such approaches do very little to improve the programme, which is the ultimate objective. For a programme as complex as the one described in the current paper, it is essential to develop rapport with implementers and make them clear about the rationale, design, activities and timeline of the evaluation, or possibly engage them in developing all these research components. Moreover, building a trusting relationship with implementers provides access to important implementation documents, timely appointment for conducting interviews, and access to implementation activities (e.g., training, meeting, CHW’ sessions at the communities) for observation.

There is a concern that concurrent evaluation is more expensive than a traditional pre–post evaluation in terms of money, time and resources. This is probably true as the duration of a concurrent evaluation is as long as the programme, and the total cost of this evaluation has exceeded 13 % of the total programme cost ($US 15·6 million). Concurrent evaluation considers several other evaluation activities, including qualitative investigation, quantitative assessment, process evaluation and economic evaluation. However, beyond just the cost of evaluation, donors/investors of a programme should assess the value of evaluation in relation to the enhanced program outcomes^(29)^. Results of this concurrent evaluation clearly demonstrate that the approach helped improve the implementation and outcomes of the intervention. Future applications may want to consider modelling the cost–benefits for more expensive evaluation designs aimed at improving programme delivery services.

## Conclusions

This paper explained the process of implementing concurrent evaluation for a real-world home fortification intervention and demonstrated the benefits of using this evaluation to improve programme implementation through real-time course correction. BRAC’s home fortification programme benefited from a series of course corrections based on the evidence generated from concurrent evaluation. Considering these experiences, we recommend using concurrent evaluation as a tool of implementation science for measuring both the implementation process and the effectiveness of a large-scale nutrition programme implemented nationally. Our evaluation approach created accountability among all programme partners. The implementation of evaluation activities, interpretation of evaluation data, and decision-making to address implementation gaps were performed with shared responsibilities among the partners. The provision of course corrections enabled the programme to expand and replicate its model in the future.

## Supplementary material

For supplementary material accompanying this paper visit https://doi.org/10.1017/S1368980020000439. click here to view supplementary material
