# Supplementary material for: Use of concurrent evaluation to improve implementation of a home fortification programme in Bangladesh: a methodological innovation
Source: Public Health Nutr. 2020 Mar 5;24(Suppl 1):s37–47. doi: 10.1017/S1368980020000439 (PMC8042578; doi:10.1017/S1368980020000439)

**Supplementary file 1. Maternal, Infant and Young Child Nutrition (MIYCN) Home-fortification Programme in Bangladesh**

BRAC implemented home-fortification of food with micronutrient powder (MNP) as part of their Maternal, Infant and Young Child Nutrition (MIYCN) Programme in Bangladesh to reduce the prevalence of iron-deficiency anaemia among children of 6-59 months. The programme was implemented nationally and targeted to reach more than 5 million children of 6-59 months through promoting the use of MNP by a cadre of female volunteer community health worker (CHW) called Shasthya Shebika (SS) and paid CHW of BRAC called Shasthya Kormi (SK); SK is also the supervisor of SS. The implementation tools included various nutritional education and awareness campaigns, MNP sale and nutrition counselling by SSs, and the creation of an enabling policy environment. A detailed home-fortification intervention under MIYCN programme are listed below.

Pushtikona (a brand name of BRAC’s MNP product) was planned to be available to BRAC’s SSs at the community level for sale among the caregivers of children in the programme areas. In addition to selling Pushtikona, the SSs of BRAC offer nutrition education and counselling to caregivers to ensure compliance with home-fortification using Pushtikona. The duration of MIYCN programme was 5 years, and this was implemented in three phases in three programme platforms. The three programme platforms of BRAC are: (i) Maternal, Neonatal, and Child Health (MNCH) Programme platform, (ii) Alive & Thrive (A&T) IYCF Programme platform, and (iii) Nutrition Programme platform. In addition to the existing intervention in each of the programme platforms, a package of interventions under the MIYCN home-fortification programme was implemented in all three platforms. The table below summarizes the existing interventions of BRAC under different platforms and additional intervention under MIYCN home-fortification programme.

| **Programme Platform of BRAC** | **Existing interventions under each platform** | **Additional interventions under MIYCN home-fortification programme in all three platforms** |
| --- | --- | --- |
| MNCH | - Provision of basic primary healthcare at the community level - Working with village health committees to motivate behaviour change in the community by addressing the issues of pregnancy, newborn and child health - Facilitating access to obstetric and newborn care at public and private health facilities | - Training (a basic training and monthly refresher training) to SSs and SKs to promote home-fortification with MNP at the household level - Provide counselling to the caregivers of children aged 6-59 months on home-fortification with MNP, monitor home-fortification activities at the community level by SSs and stimulate SSs and SKs with the provision of incentives to promote MNP at the community level. - Supportive supervision for the SSs by SKs, Programme Organizer and Officer-Supply Chain and Quality Assurance - Demand-generation activities included social and behaviour change communication through various channels (i.e mass media campaign, social mobilization) to sensitize the community and gatekeepers to increase demand for MNP. - Interventions for creating enabling environment for home-fortification with MNP included advocacy to the national-level key stakeholders towards enabling policy, building government leadership and buy-in on home- fortification with MNP, and aligning and ensuring that key stakeholders and civil society support and demand home-fortification as part of optimum IYCF |
| Alive and Thrive | - Home-visit by the CHWs to provide IYCF counseling to mothers, coaching, demonstration, problem-solving, and reference - Antenatal care sessions and postnatal care visits by the CHWs for early initiation of breastfeeding and exclusive breastfeeding, and providing support for good positioning and attachment - Health forums where the CHWs disseminate IYCF messages and discuss issues in small groups with pregnant women, mothers, and family members |  |
| Nutrition | - CHWs and nutrition promoters visit households in their communities and provide counselling, coaching, and demonstration for creating awareness about nutrition - CHWs create awareness about nutrition of adolescent girls and encourages mothers and family members about many issues, like intake of healthful and various types of food, early initiation of breastfeeding, exclusive breastfeeding till 6 months, breastfeeding for at least two years, and initiation of complementary feeding after six months |  |

***Programme areas and implementation phases:*** Bangladesh is administratively divided into 8 divisions, 64 districts, and 492 sub-districts. The sub-districts are the third-lowest tier of regional administration in Bangladesh. In Year 1, starting in January 2014, BRAC had started up the MIYCN project in the 68 Maternal, Neonatal, and Child Health (MNCH) programme sub-districts (in 10 districts) and 2 urban slums in Dhaka. In Year 2 (January 2015), the programme had been expanded to 48 Alive & Thrive programme sub-districts (in 15 districts) and 2 urban slums in Dhaka. Finally, in Year 3 (in 2016), the programme covered the 48 Nutrition Programme sub-districts under BRAC (in 9 districts) and 2 urban slums in Dhaka to reach a total of 164 sub-districts and 6 urban slums with the home-fortification intervention. Although Dhaka did not belong to any platforms (i.e. MNCH, A&T, and Nutrition) of BRAC, when the intervention under MIYCN programme started at different time points (Year 1, Year 2, Year 3) at different platforms, BRAC included 2 urban slums from Dhaka simultaneously in each time-point. The catchment area of an SS has been defined as a BRAC community and each community consists of 150 to 500 households. On an average, each sub-district has 100-200 BRAC communities. A sub-district-wise map of Bangladesh highlighting the home-fortification programme areas and implementation phases is presented below.


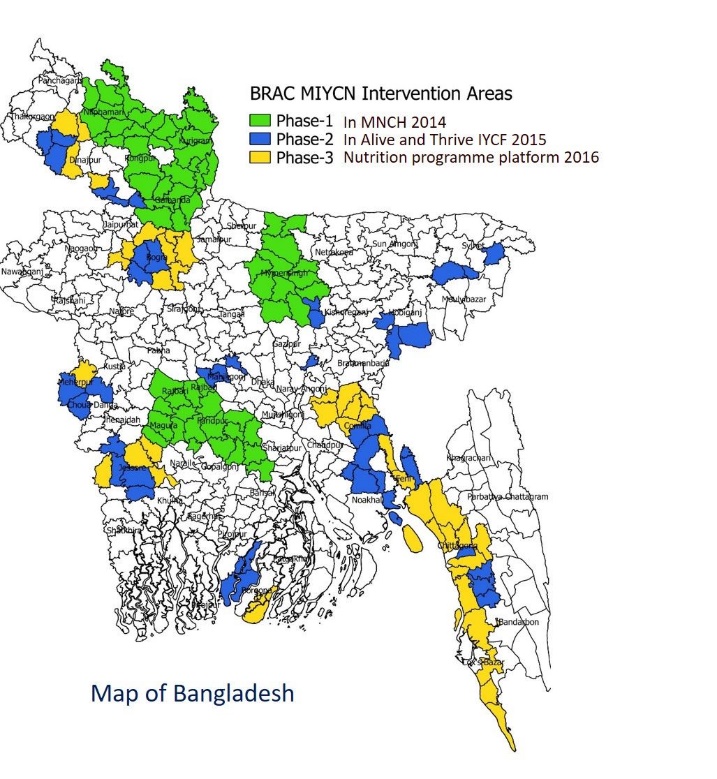

Supplement: Supplementary file 1 [file S1368980020000439sup001.docx]
